# Cancer-associated fibroblasts in osteosarcoma: key players in immune escape and targeted therapy

**DOI:** 10.3389/fimmu.2025.1668535

**Published:** 2025-09-01

**Authors:** Junjie Fan, Yujie Jin, Feng Lv, Weidong Wu, Li Sun, Churong Wang

**Affiliations:** Department of Orthopedics, Suzhou Ninth People’s Hospital, Soochow University, Suzhou, Jiangsu, China

**Keywords:** osteosarcoma, cancer-associated fibroblasts, immune evasion, therapy resistance, tumor microenvironment

## Abstract

Osteosarcoma represents the most common principal malignant bone tumor that predominantly appears among teenagers and children. While multimodal treatment methods have greatly evolved with time, survival for recurrent or metastatic disease remains low due to the resistance that accumulates during treatment. Increasing evidence identifies the tumor microenvironment (TME), in particular cancer-associated fibroblasts (CAFs), as playing an important role in imposing immune suppression, enhancing tumor aggressiveness, and mediating resistance toward immunotherapy and chemotherapy. This article gives an overview of the derivation, phenotypic heterogeneity, and mechanisms of action of CAFs during osteosarcoma, such as facilitating immune escape, survival signaling, drug efflux, regulation of genes through exosomes, and inhibiting ferroptosis. Furthermore, we present existing and new treatment methods that are centered on CAFs, such as suppression of the paracrine pathway (e.g., IL-6/STAT3, TGF-β), depletion of CAFs lineages by targeting fibroblast activation protein (FAP), and conversion toward tumor-restraining CAFs. Other methods that are gaining popularity are targeting CAFs-releasing exosomes and metabolic liabilities. By shedding light on CAFs-based methods for imposing resistance and trying targeted treatments, this review offers insights into novel therapeutic combinations that can overcome treatment barriers and improve survival outcomes in osteosarcoma regimens.

## 1 Introduction

Osteosarcoma is the most common primary malignant bone tumor and occurs most frequently in adolescents and young children. While aggressive multimodality treatment—neoadjuvant chemotherapy combined with limb-sparing surgery and adjuvant chemotherapy—has improved patient survival for patients with localized tumors, patients with recurrent or metastatic tumors remain poor risks, with 5-year survival rates of under 30% (1, 2). Therapeutically, despite thorough investigative efforts during three decades, there has been scant meaningful breakthrough.

Recalcitrance of classic and immune therapies for osteosarcoma is progressively more controlled by the contribution of the TME, and TME plays a significant role in immune escape, resistance to chemotherapy and radiation, and development of metastasis (3). As a part of non-malignant stromal elements, cancer-associated fibroblasts (CAFs) are found to play a crucial role in these events. Initially considered as static supporters, CAFs are currently found to be active cells that possess the capacity to remodel the extracellular matrix (ECM), suppress immune responses, and facilitate therapy resistance (4–6). Although CAFs has been widely studied in epithelial solid tumors such as breast cancer, pancreatic cancer and colorectal cancer, its specific function and mechanism in osteosarcoma are still lack of systematic elucidation. This knowledge gap limits our in-depth understanding of CAFs-mediated interstitial remodeling and its unique role in the pathogenesis and drug resistance of osteosarcoma. Therefore, clarifying the role of CAFs in osteosarcoma is of great significance for finding new therapeutic targets and improving the therapeutic effect of this invasive bone malignancy (4, 5, 7).

CAFs are abundantly present in both primary and metastatic osteosarcoma lesions and are associated with unfavorable outcome and immunosuppressive microenvironment (8). Due to their link with immune cells, tumor stem-like cells, and endothelial cells, they create a multi-potent crosstalk that promotes tumor growth. However, CAFs also secrete cytokines such as TGF-β, IL-6, and CXCL12 that not only modulate immunity but are implicated in chemoresistance and failure of immune checkpoint blockade (9, 10).

By virtue of these pleiotropic roles, CAFs would represent a new therapeutic target for immune escape breaking and treatment resistance in osteosarcoma. The current review hopes to collate available information pertaining to CAFs in this neoplasm with a concern for their role in immune evasiveness and treatment resistance, and discuss new strategies for therapeutically targeting such processes.

## 2 Origins and characteristics of CAFs in osteosarcoma

CAFs are highly active stromal cells of the TME that support tumor growth, immune evasion, and drug resistance. Although nearly everything that is understood regarding CAFs originates in epithelial cancers, there is recent literature available that highlights their equally significant role in mesenchymal cancers such as osteosarcoma (11, 12).

### 2.1 Origins of CAFs in bone tumors

CAFs can arise from multiple cellular sources, including tissue-resident fibroblasts, pericytes, bone marrow–derived mesenchymal stem cells (MSCs), and endothelial cells undergoing endothelial-to-mesenchymal transition (EndMT) (13).

In the bone metastatic microenvironment, TGF-β reshapes the niche by modulating osteoclasts, osteoblasts, and fibroblast-like stromal cells, thereby promoting tumor cell colonization. Its recruitment and activation of stromal fibroblasts contribute to establishing a pro-metastatic and immunosuppressive milieu (5, 14).

Activated CAFs exhibit a secretory and contractile phenotype with enhanced production of ECM and secretion of immunomodulatory and survival factors. These characteristics are generally maintained through persistent paracrine signaling and epigenetic remodeling (10).

### 2.2 Phenotypic markers and functional subtypes

CAFs cells do not share one common marker. They are often identified by α-smooth muscle actin (α-SMA), FAP, and platelet-derived growth factor receptors (PDGFRα/β) (15). Immunohistochemical examination identified multiple FAP^+^ and α-SMA^+^ fibroblasts in primary osteosarcoma lesions and lung metastatic nodules (7, 16, 17). Single-cell transcriptomics defined that CAFs are not a homogeneous population. At least two dominant subtypes are invariably observed: (1) myofibroblastic CAFs (myCAFs): with strong α-SMA expression, with ECM remodeling gene patterns and contractility; (2) inflammatory CAFs (iCAFs): with enrichment for cytokines and chemokines IL-6, CXCL12, and CCL2 (18–20). These types could both be found within tumors and possibly alternate each other according to signals by cancer and immune cells, leading to their functional plasticity.

## 3 CAFs-mediated immune escape in osteosarcoma

One of the major obstacles to successful cancer immunotherapy is the immunologically “cold” tumor microenvironment, where there is minimal infiltration by cytotoxic T cells and there are abundant immunosuppressive signals (11). CAFs are central characters in plotting such an immune-excluded phenotype. Through chemokines, cytokines, and extracellular matrix components, CAFs mold the immune context and enable tumor cells to escape host immune detection (15).

### 3.1 Suppression of T cell infiltration and function

In osteosarcoma, CAFs secrete CXCL12, which can bind to CXCR4 on the surface of T cells, hindering their infiltration into the tumor core area (21). This effect forms an “immune shell” around the tumor, physically excluding T cells from contact with tumor cells. Secondly, TGF - β produced by CAFs significantly weakens T cell function, inhibits its proliferation, cytotoxicity, and cytokine production, while promoting the differentiation of initial CD4 ^+^ T cells into immunosuppressive regulatory T cells (Tregs). These processes collectively establish an immunosuppressive tumor microenvironment, promoting cancer progression and enhancing resistance to treatment (10, 22).

### 3.2 Recruitment of immunosuppressive immune cells

In addition to directly inhibiting effector T cells, CAFs also recruit multiple immune suppressive cell populations by secreting chemokines such as CCL2, IL-6, and CXCL8 (5). These factors attract bone marrow-derived suppressor cells (MDSCs), M2 polarized macrophages, and Tregs, synergistically creating an inhibitory immune microenvironment (23). For example, IL-6 mediated activation of the JAK/STAT3 pathway has been shown to be closely associated with enhanced immune suppression and accelerated tumor development in osteosarcoma (24).

### 3.3 ECM-mediated immune exclusion

Cancer associated fibroblasts promote immune escape by reshaping the ECM, which increases matrix density and stiffness, forming a physical barrier that hinders the infiltration and migration of cytotoxic T lymphocytes (10). In the microenvironment of osteosarcoma, ECM components such as type I collagen and fibronectin are significantly elevated, while the expression of matrix metalloproteinases (MMPs) is also upregulated (25). These changes not only hinder the entry of immune cells into the tumor area, but are also associated with poor response to immune checkpoint inhibitors, further highlighting the role of ECM remodeling in immune suppression of osteosarcoma (26).

### 3.4 Metabolic reprogramming and immune dysfunction

The latest research shows that CAFs alter the metabolic characteristics of the TME by secreting lactate and acidifying the extracellular environment (27). This metabolic change can inhibit T cell effector function and promote immune tolerance (28). In addition, extracellular vesicles derived from CAFs may carry immunosuppressive miRNAs and proteins that can regulate the phenotypes of dendritic cells and macrophages (29). Similar phenomena have also been observed in osteosarcoma: fibroblasts exhibit high glycolytic properties, while lactate acts as a “metabolic signal” that promotes epithelial mesenchymal transition (EMT) of tumor cells and activates immunosuppressive pathways (30).

## 4 CAFs-mediated therapy resistance in osteosarcoma

Treatment resistance is the main reason for the failure of osteosarcoma treatment, especially in cases of recurrence and metastasis. Although genetic mutations and epigenetic adaptations of tumor cells are known resistance mechanisms, increasing evidence suggests that the TME-especially CAFs-plays a central role in chemotherapy, immunotherapy, and even radiotherapy resistance. CAFs contribute to treatment failure through various mechanisms such as paracrine signaling, ECM remodeling, metabolic support, and tumor stemness maintenance (31).

### 4.1 Paracrine signaling activation and survival pathways

As the main matrix component in the microenvironment of osteosarcoma, CAFs secrete a large amount of soluble factors such as IL-6, TGF-β, and CXCL12 (also known as SDF-1). Recent studies demonstrate that cytokines secreted by CAFs—such as IL−6, IL−11, TGF−β, and growth differentiation factor—can activate several oncogenic survival pathways in tumor cells. Moreover, emerging evidence highlights that CAFs can also suppress ferroptosis, a novel form of regulated cell death central to anticancer therapy. In osteosarcoma, FSP1 has been identified as a key determinant of cellular susceptibility to ferroptotic death, where high FSP1 expression confers resistance, while its inhibition markedly sensitizes tumor cells to ferroptosis-inducing agents (32). Similarly, in pancreatic ductal adenocarcinoma, CAFs secrete cysteine via a TGF−β/SMAD3/ATF4–dependent transsulfuration pathway, enhancing glutathione (GSH) synthesis in tumor cells and thereby blocking lipid peroxidation–driven ferroptosis (33). Mechanistically, hyperactivation of the PI3K/AKT axis in cancer cells is also known to inhibit ferroptosis by modulating downstream regulators such as SLC7A11, GPX4, NRF2, and iron metabolism components (34). Taken together, these observations support the hypothesis that CAFs−mediated metabolic rewiring and signaling crosstalk may contribute to ferroptosis resistance and therapy failure in osteosarcoma.

### 4.2 Enhancement of drug efflux via ABC transporters

ABC transporters are a large class of transporters that rely on ATP (hydrolysis) for energy to transport various substrates across the membrane. In osteosarcoma, a key mechanism by which CAFs mediate therapeutic resistance is through induction of ABCB1/P−glycoprotein in tumor cells, leading to active efflux of chemotherapy agents (35, 36). Also, a recent study demonstrated that the glycosyltransferase C1GALT1, which can be upregulated in the tumor stroma, plays a critical role in promoting doxorubicin resistance by inducing ABCC1 expression in osteosarcoma cells (37). Furthermore, targeted disruption of ABCB1 using CRISPR/Cas9 has been shown to restore doxorubicin sensitivity in resistant osteosarcoma cell lines (e.g., KHOSR2, U-2OSR2), further confirming the role of CAFs-induced signaling in drug resistance (35).

### 4.3 Exosome-mediated gene regulation and miRNA transfer

Recent studies suggest that tumor-derived exosomes not only remodel the pre-metastatic niche but also modulate stromal cells to support tumor progression and drug resistance. For instance, osteosarcoma-secreted exosomal linc00881 can be internalized by lung fibroblasts, inducing their transformation into CAFs-like phenotypes (38). And once activated, CAFs can further contribute to tumor progression and therapy resistance by releasing their own exosomes enriched with oncogenic non-coding RNAs and proteins. These CAFs-derived exosomes can be internalized by osteosarcoma cells and reprogram gene expression to enhance survival pathways—such as PI3K/AKT—and suppress apoptotic responses (39, 40). For instance, exosomal miR-1228 derived from cancer-associated fibroblasts has been shown to promote osteosarcoma cell migration and invasion by directly targeting the tumor suppressor SCAI. This exosome-mediated intercellular communication reinforces a pro-tumorigenic microenvironment and may contribute to the development of aggressive and potentially chemoresistant phenotypes in osteosarcoma (41).

## 5 Targeting cancer-associated fibroblasts in osteosarcoma

**Figure 1:**
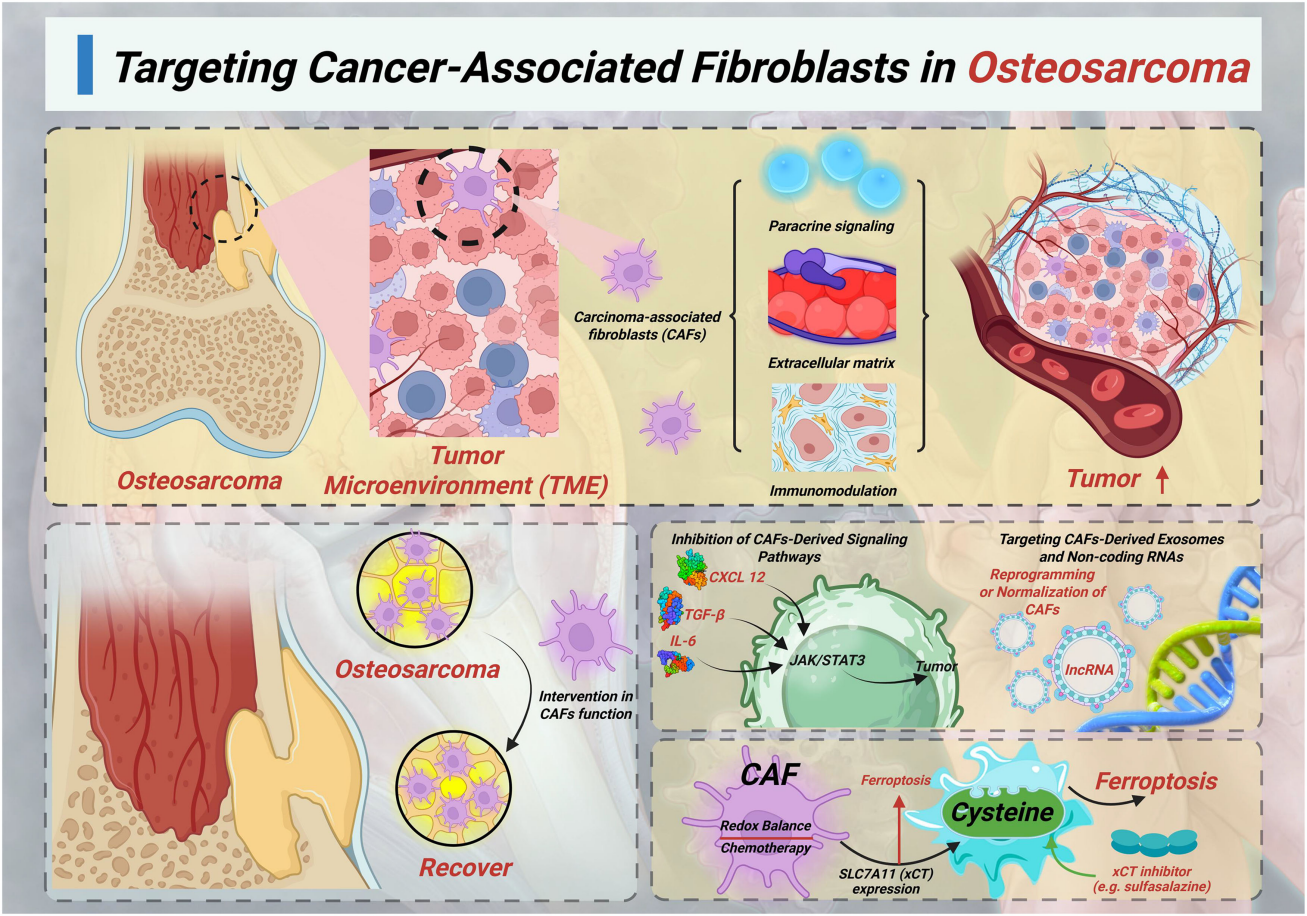
Targeting cancer-associated fibroblasts in osteosarcoma.

CAFs are one of the most abundant and active components of the TME in osteosarcoma (**Figure 1**). Although they are not inherently malignant, CAFs promote tumor progression through mechanisms such as paracrine signaling, ECM remodeling, immune regulation, and metabolic support. In osteosarcoma, they promote drug resistance in tumor cells by activating survival signaling pathways, inhibiting ferroptosis, enhancing drug efflux, and promoting immune escape. It also forms physical and chemical barriers around the tumor, hindering drug delivery (6, 42).

Due to their high genetic stability, CAFs are less likely to develop drug-resistant mutations compared to tumor cells, making them a promising therapeutic target. Intervention in CAFs function is expected to enhance the efficacy of chemotherapy and immunotherapy by disrupting the mechanisms that support tumors (5).

### 5.1 Inhibition of CAFs-derived signaling pathways

One main strategy for targeting CAFs is to block its signaling pathway activated in osteosarcoma cells. CAFs can secrete various soluble cytokines and growth factors, including IL-6, TGF - β, and CXCL12, which bind to receptors on tumor cells and activate oncogenic signaling pathways such as JAK/STAT3, PI3K/AKT, and MAPK. Among them, the IL-6/STAT3 axis is particularly crucial in promoting resistance to amphotericin B and Cisplatin, by enhancing survival signaling and anti-apoptotic ability (4, 42). The treatment methods for blocking this axis include IL-6 receptor antibodies (such as tocilizumab), STAT3 small molecule inhibitors, or JAK kinase inhibitors, which are expected to reverse chemotherapy resistance induced by CAFs. Similarly, TGF - β signaling plays an important role in EMT, tumor stemness, and matrix deposition. Small molecule inhibitors such as Galunisterib (LY2157299) target TGF - β type I receptors and have been studied in various solid tumors, particularly in the CAFs rich microenvironment (43–45).

### 5.2 Direct targeting and depletion of CAFs populations

Another strategy is to physically or functionally clear CAFs by targeting surface markers or lineage specific proteins. FAP is selectively overexpressed in CAFs and osteosarcoma cells, but shows minimal expression in normal fibroblasts (46, 47). In osteosarcoma, FAP has been shown to promote tumor progression by enhancing angiogenesis through activation of the AKT and ERK signaling pathways, and by facilitating cell proliferation, migration, and invasion (48). These findings not only support its role as a prognostic marker associated with poor outcomes (49). Although direct *in vivo* evidence in osteosarcoma is limited, the presence of FAP ^+^ CAFs is associated with poorer prognosis and stronger drug resistance. Therefore, the use of anti FAP strategies in combination with cytotoxic drugs may improve therapeutic efficacy (50). Therefore, integrating FAP-targeted strategies with conventional chemotherapy may offer a synergistic approach to overcome resistance and improve clinical outcomes in osteosarcoma (47, 48).

### 5.3 Reprogramming or normalization of CAFs

In addition to direct clearance, recent studies have also attempted to reprogram activated CAFs into a resting state or anti-tumor phenotype. In osteosarcoma models, recent studies have begun to explore reprogramming activated CAFs into a quiescent or tumor-suppressive phenotype. Vitamin D (via VDR activation) was shown to inhibit EMT and ROS (reactive oxygen species) signaling—key drivers of metastasis and tumor survival—and thus may indirectly modulate stromal fibroblasts toward a less tumor-supportive state in osteosarcoma xenograft models (51). Although direct data on ATRA(All-Trans Retinoic Acid) in osteosarcoma CAFs is still lacking, preclinical evidence from pancreatic and other cancers supports the concept that vitamin A derivatives can normalize CAFs and suppress their pro−tumor secretome (52). And the use of HDAC (histone deacetylase) inhibitors or BET(Bromodomain and Extra-Terminal domain) inhibitors for epigenetic remodeling is also expected to transform CAFs into a low tumorigenic state (4). However, studies on the application of ATRA and HDAC inhibitors in osteosarcoma remain limited, so further research is needed to evaluate their therapeutic potential in this context.

### 5.4 Targeting CAFs-derived exosomes and non-coding RNAs

CAFs also plays a role in remote regulation by releasing extracellular vesicles (EVs), which contain miRNA, lncRNA, and proteins. In osteosarcoma, CAFs have been shown to release EVs enriched in non−coding RNAs—particularly miRNAs and lncRNAs—that modulate tumor cell behavior. For instance, CAFs−derived exosomal miR−21−5p promotes OS cell proliferation and chemoresistance by targeting PIK3R1, thereby activating the PI3K/Akt/mTOR pathway (53, 54). Similarly, exosomal lncRNA−SNHG17 transported from CAFs acts as a competing endogenous RNA (ceRNA) to sponge miR−2861, leading to MMP2 upregulation, enhanced migration and invasion of osteosarcoma cells (54). Additionally, lncRNA PVT1—abundant in exosomes from bone marrow–derived mesenchymal stromal cells—has been shown to promote metastasis via the miR−183−5p/ERG axis, and may likewise be relevant in CAFs−EV cargos (53). These non-coding RNAs often dysregulate key signaling nodes such as PTEN/PI3K/AKT under chemotherapy pressure, fostering apoptosis resistance and aggressive phenotypes. Although direct CAFs-specific PVT1 data in OS is still emerging, the functional parallels with SNHG17 and miR-21 support a convergence on PI3K/Akt signaling modulation (53, 55). And potential intervention strategies targeting these EVs include: (1) using drugs such as GW4869 or sphingomyelinase inhibitors to suppress their generation or secretion; (2) blocking osteosarcoma cell uptake of EVs; (3) selectively targeting pathogenic RNA cargo, for example, using antisense oligonucleotides to interfere with SNHG17 or miR-21, to disrupt cancer-promoting EV signaling while maintaining normal stromal cell communication.

### 5.5 CAFs-mediated suppression of ferroptosis and metabolic interventions

The latest research has found that CAFs can inhibit ferroptosis by regulating iron metabolism, promoting glutathione synthesis, and upregulating SLC7A11 (xCT) expression in tumor cells. CAFs helps tumor cells resist oxidative stress induced by chemotherapy by maintaining redox balance (33). Although direct experimental validation in osteosarcoma is currently insufficient, targeting xCT mediated cysteine uptake with inhibitors such as sulfasalazine can make tumor cells more sensitive to ferroptosis inducers. This strategy is expected to overcome drug tolerance dominated by CAFs by disrupting redox homeostasis and metabolic support, especially in tumors with high CAFs activity and strong antioxidant stress resistance (56).

## 6 Conclusion and future perspectives

For orthopedic surgeons, osteosarcoma remains a major challenge in both clinical treatment and research. Although numerous studies have clearly supported the tumor promoting effect of CAFs in osteosarcoma, especially in immune escape and treatment resistance, we should also recognize that the function of CAFs is context dependent and may even have contradictory manifestations. In other solid tumors (such as pancreatic cancer and colorectal cancer), some CAFs subgroups (such as Meflin^+^ CAFs) have been confirmed to have tumor inhibitory potential, which can inhibit tumor progression, maintain matrix softness, promote drug delivery and enhance the response of immunotherapy. However, there is no systematic study on the role of Meflin^+^ CAFs or other tumor suppressive CAFs in osteosarcoma. Therefore, in-depth exploration of the functional characteristics of different CAFs subgroups in osteosarcoma will help to comprehensively understand its two-way regulatory role in tumor occurrence and development, and provide theoretical basis and potential treatment strategies for accurately targeting CAFs (3, 8, 26, 57, 58).

In osteosarcoma, CAFs has been proved to promote tumor progression and chemotherapy resistance through a variety of mechanisms. Studies have found that NgR modified CAFs derived exosomes can deliver circ_0004872-109aa small peptides, which can effectively reverse the tolerance of osteosarcoma to chemotherapy drugs by promoting autophagy-dependent ferroptosis (59). In addition, studies have pointed out that IL-6 from CAFs or MSc can activate STAT3 signaling pathway, thereby enhancing the resistance of osteosarcoma cells to doxorubicin, cisplatin and other drugs (43). At the same time, the exosomes containing IL-6 and IL-8 secreted by osteosarcoma cells can induce normal fibroblasts to transform into CAFs, further forming a feedback loop, helping tumor malignant progression and drug resistance (53). These studies show that although CAFs itself is not a malignant cell, its tumor promoting properties make it a potential therapeutic target. However, CAFs population itself has a high degree of heterogeneity and functional plasticity, which poses a major challenge to targeted therapy. More importantly, studies have found that excessive clearance of CAFs may have adverse effects, such as promoting tumor invasion or inducing immune dysfunction. Therefore, future treatment strategies need to be more precise and context specific.

Moreover, integrating multimodal technologies such as single-cell omics, spatial transcriptomics, and advanced imaging will provide us with key means to comprehensively understand CAFs function at the spatial scale (18). At the same time, combining CAFs targeted drugs with immunotherapy or programmed cell death inducers is expected to overcome existing resistance barriers and achieve synergistic efficacy. Therefore, a deeper understanding of the interrelationships between CAFs and other components of TME will open up new paths for precision medicine exploration of osteosarcoma, which may change the treatment pattern of refractory or metastatic patients.
